# Electrons and Their Multiple Kinetic Fates in an Ionic Liquid

**DOI:** 10.1021/jacs.5c07005

**Published:** 2025-06-27

**Authors:** Hung H. Nguyen, Katie Huber, Dishan Das, Bichitra Borah, Matthew S. Emerson, Meghan Knudtzon, James F. Wishart, David A. Blank, Claudio J. Margulis

**Affiliations:** † Department of Chemistry, 4083 The University of Iowa, Iowa City, Iowa 52242, United States; ‡ Department of Chemistry, University of Minnesota, Minneapolis, Minnesota 55455, United States; ¶ Chemistry Department, 8099 Brookhaven National Laboratory, Upton, New York 11973, United States

## Abstract

Ionic liquids (ILs) for electrochemical, nuclear, and solar energy applications operate under harsh conditions, where electrons and transient radical species can form. This communication discusses why anions such as bis­(trifluoromethylsulfonyl)­imide (Tf_2_N^–^) are reduced at the electron-rich electrode whereas in laser photoionization or pulse radiolysis studies, where electrons are ejected from species in the bulk, we often detect long-lived electrons in cavities that interact with IL cations instead. This work argues that bulk excess electrons generated photolytically or radiolytically follow kinetically favored pathways. As such, cavity electrons may not be the most energetically favorable states, but when they form, and they do form, they are kinetically stable. Reduction reactions of anions or electron localization in cavities and subsequent reactions are all expected outcomes. Here we focus on a pyrrolidinium-based IL of the dicyanamide (N­(CN)_2_^–^) anion because of its large electrochemical window and very low viscosity, which are ideal for energy applications.

## Graphical abstract

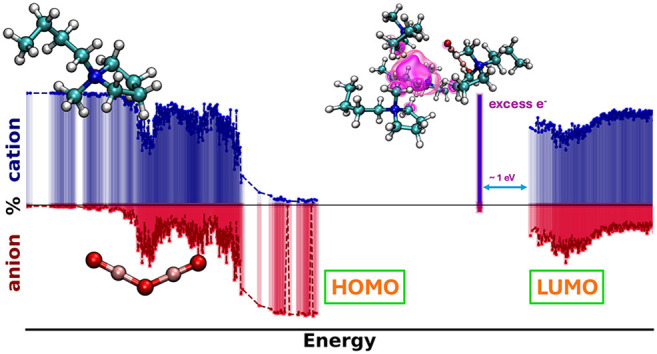

To rationalize the very complex behavior of an excess electron in ionic liquids, we must consider the following facts: (i) even the simplest ILs are made of two components, (ii) each of these components is charged, and (iii) each has its own condensed-phase HOMO–LUMO gap. Yet, condensed-phase cationic and anionic HOMO–LUMO gaps are not independent, and consistent with this, polarization and some degree of charge transfer are expected; ILs are also nanostructured and have apolar domains. The more practically important quantities are the experimentally observable band gap of the IL and its closely related electrochemical window, which depend on the energetic alignment of cationic and anionic HOMO–LUMO gaps in the condensed phase or at interfaces. *As a rule of thumb, (1) the more electronically insulating an IL is (or the larger its band gap, or the larger its electrochemical window), and (2) the more ionically conductive it is (often linked to low viscosity), the better for application purposes.* Because of their advantageously low viscosity and often excellent electronically insulating properties (large electrochemical windows), pyrrolidinium-based ILs of the Tf_2_N^–^ and N­(CN)_2_^–^ anions are particularly popular, and in this work, we focus on an excess electron in 1-butyl-1-methylpyrrolidinium dicyanamide ([Pyrr_1,4_]­[N­(CN)_2_]).^1–3^ A curious puzzle, at least 20 years in the making, is the focus in electrochemistry on anionic reactions at the electron-rich electrode (anionic reduction products),^4–10^ whereas photochemistry and radiation chemistry studies^11–18^ on the same or similar liquids highlight the very stable nature of cavity electrons solvated by cations. For example, the electrochemical reduction of Tf_2_N^–^, $N{\left({\mathrm{SO}}_{2}{CF}_{3}\right)}_{2}^{-}+e^{-}\to N{\left({\mathrm{SO}}_{2}{CF}_{3}\right)}_{2}^{2-\cdot }$ , with fast subsequent cleavage of the anion, can be followed at the electron-rich electrode as a function of the number of chronoamperometric cycles via IR spectroscopy.^4^ Quantum calculations on excess electrons in [Pyrr_1,4_]­[N­(CN)_2_] and [Pyrr_1,4_]­[Tf_2_N] or similar systems have supported this electrochemistry-style view that reduction of such anions is energetically downhill and highly favorable in the gas and bulk phases.^4,19,20^ So which one is it? Ultrafast downhill reduction of anions or quite stable cavity electrons that are solvated by cations with which they can interact and also react? And, how can these particle-in-a-box type states live hundreds of nanoseconds in pyrrolidinium-based ILs of Tf_2_N^–^ or N­(CN)_2_^–^ when anionic reduction is available and downhill and anions are everywhere?

Figure 1 shows computational (TDDFT) and experimental (optical pump–probe transient absorption and pulse radiolysis) spectra of the cavity electron in [Pyrr_1,4_]­[N­(CN)_2_] (for methodology details, see Sections S.1.1 and S.1.2, as well as the figure caption). At very short sub-picosecond times, the NIR spectrum is very broad; in fact, the optical pump–probe version is essentially flat over its limited energy range (solid red line). Some of the low-energy transitions observed computationally at very short time simply move the cavity electron around, and this is indicative of a dry electron that can exist in many parts of the liquid and has no strong preference for a specific location; in fact, we use this lack of localization preference to create an ansatz for initial conditions in our DC-r^2^SCAN^21,22^ trajectories (see Section S.1.2).

**Figure 1:**
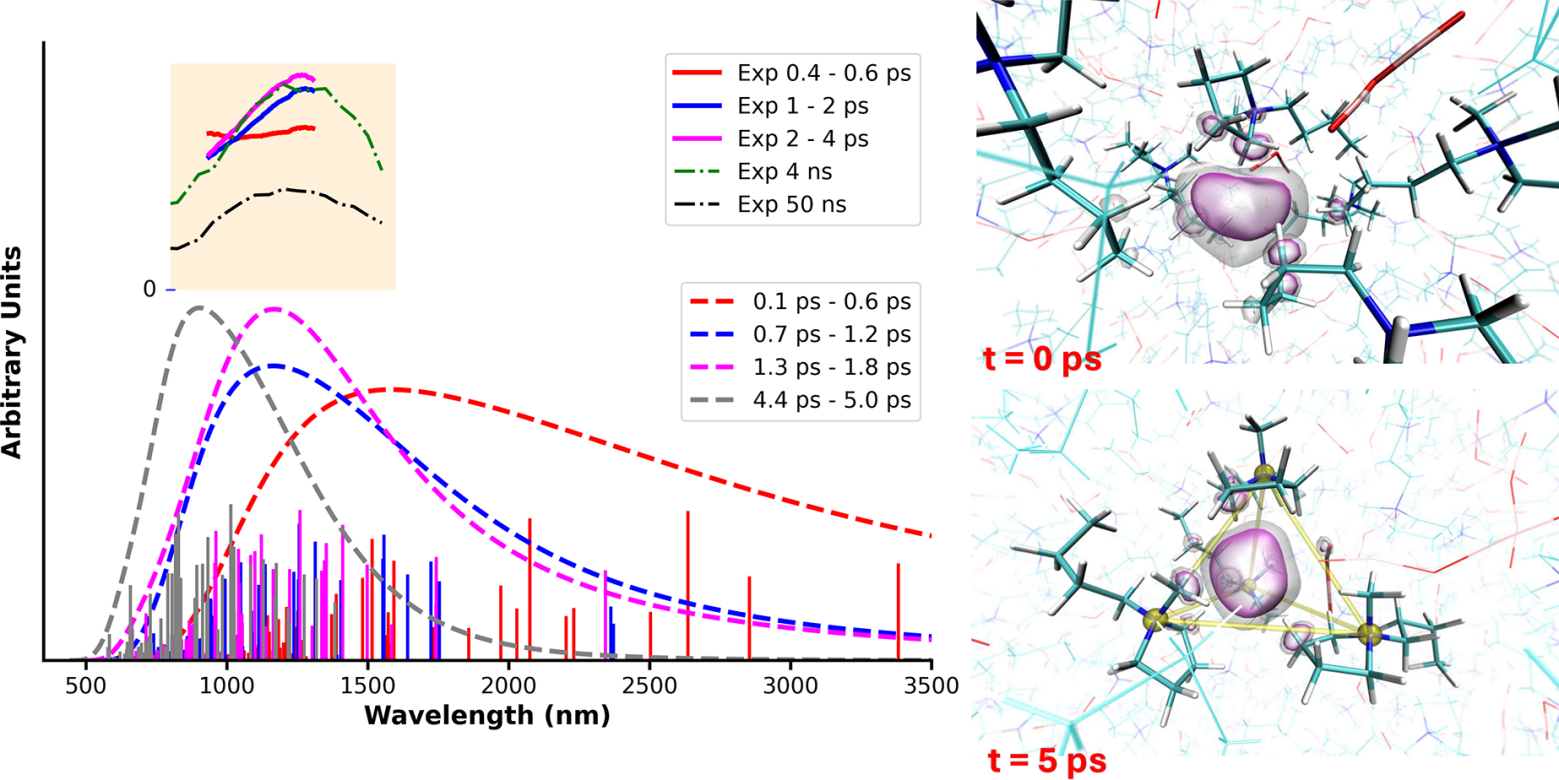
(Left) From trajectories described in the methodology section using the DC-r^2^SCAN method, TDDFT spectra using PBE0-D3 with 50% HFX (vertical lines of a given color, tagged in the bottom figure label box, are oscillator strengths of 5 spectra separated by 0.1 ps and include the lowest 10 excitations). Associated with these vertical lines are dashed lines corresponding to the broadened version of the combined line spectra, which are there for visual effect only; broadening was applied in the eV scale and then converted to wavelength. In the orange background panel, located above the computational results, are (1) the time-resolved pump–probe data (solid lines) collected for ref 11 and averaged over the time period indicated in the top figure label box and (2) longer-time pulse radiolysis spectra (dot-dash lines) highlighting that a cavity electron is long-lived in this IL. (Right) Snapshots of the cavity electron at 0 and 5 ps corresponding to the same simulations; the electron is represented by density isosurfaces, and the yellow lines at 5 ps connect the nitrogens in each of four cations solvating the excess electron. Notice how at time zero the electron is surrounded by both cations and anions; at later time, due to solvent relaxation and reorganization, it is mostly cations that solvate the electron, and these point their charged heads toward it.

At later times, the spectrum of the cavity electron narrows and moves to higher energy (lower in nm). This is indicative of deeper traps created by solvent reorganization; the full process of solvation and the lifetime of cavity electrons in [Pyrr_1,4_]­[N­(CN)_2_] are much longer than our simulations. We can see this from our pulse radiolysis data at 4 and 50 ns, which still clearly show the particle-in-a-box style spectra characteristic of cavity electrons. Notice from the time-stamped simulation snapshots in Figure 1 that at 5 ps the electron localized in a cavity is already fully surrounded by cations, and these point their charged portions in its direction; this is not the case at time zero. Consistent with this process of electron solvation, the left column in Figure 2 shows a decreasing singly occupied molecular orbital (SOMO) energy as a function of time and orbital participation (denoted as changes in charge) of several cations (the range going from 2 to 4) next to the electron.

**Figure 2:**
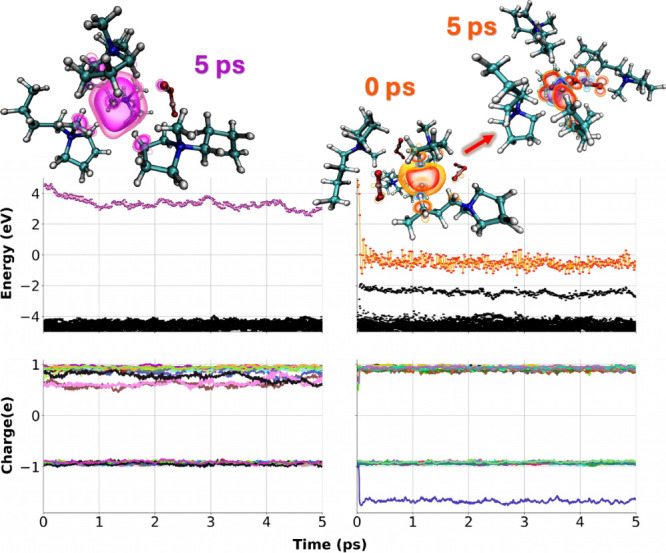
Top graphs show energy diagrams as a function of time and bottom graphs the Lowdin charge of the different ionic species for (left) a trajectory that results in cavity localization and (right) one that results in anionic localization of the excess electron forming N­(CN)_2_^2–·^; notice the charge of the reduced anion approaching a value of −2. In the top panel the SOMO is highlighted with color; in the bottom panel, each ion is depicted with a color.

The right panel in Figure 2 and overlaid simulation snapshots depict a very different story, one in which a different ansatz generated a cavity electron that on a sub-picosecond time scale found and reduced a N­(CN)_2_^–^ anion, resulting in the N­(CN)_2_^2–·^ radical. The reader should notice that the SOMO energy drops significantly upon this initial reduction of the anion and also that the energy of N­(CN)_2_^2–·^ is several eVs below that of a cavity electron. Clearly, the localization on an anion is the more energetically favorable of the two options, but that does not mean that the electron will always take the anion reduction pathway.

To put the DC-r^2^SCAN results into context with the more traditional but significantly more expensive PBE0-D3 approach using different amounts of HFX,^23^ Figure 3 shows a grid of trajectory results that probe the effects of (1) the amount of HFX and (2) the ansatz for electron localization. In these simulations, at time zero, we removed from [Pyrr_1,4_]­[N­(CN)_2_] a single special anion^24^ of varying size (F^–^, Cl^–^, BF_4_^–^) that we had initially swapped into each system with the intention of having the electron localize where the removed anion was formerly located, in a way, “pre-solvating” it. Independent of the initial ansatz, when using 50% HFX, the electron localizes as a cavity that begins to undergo solvation (see the third column in Figure 3). The energetics of the SOMO in the third column (50% HFX) are consistently similar to the DC-r^2^SCAN results when the electron is localized in a cavity. Recall from Figure 1 that those results match the spectroscopic observations quite well. As we reduce the amount of HFX and concomitantly allow the electron to be more delocalized, it becomes more reactive, or at least more reactive on the time scale of observation. At 25% HFX, the typical value for nominal PBE0, in one trajectory, we see the excess electron, initially localized as a cavity, quickly attacking a Pyrr_1,4_^+^ cation, rendering 1-butylpyrrolidine and the CH_3_^·^ radical. In another trajectory, we see the formation of N­(CN)_2_^2–·^; the same radical also forms in one of the 40% HFX examples. The two other 40% HFX trajectories render, on the time scale of observation, the stable cavity electron.

**Figure 3:**
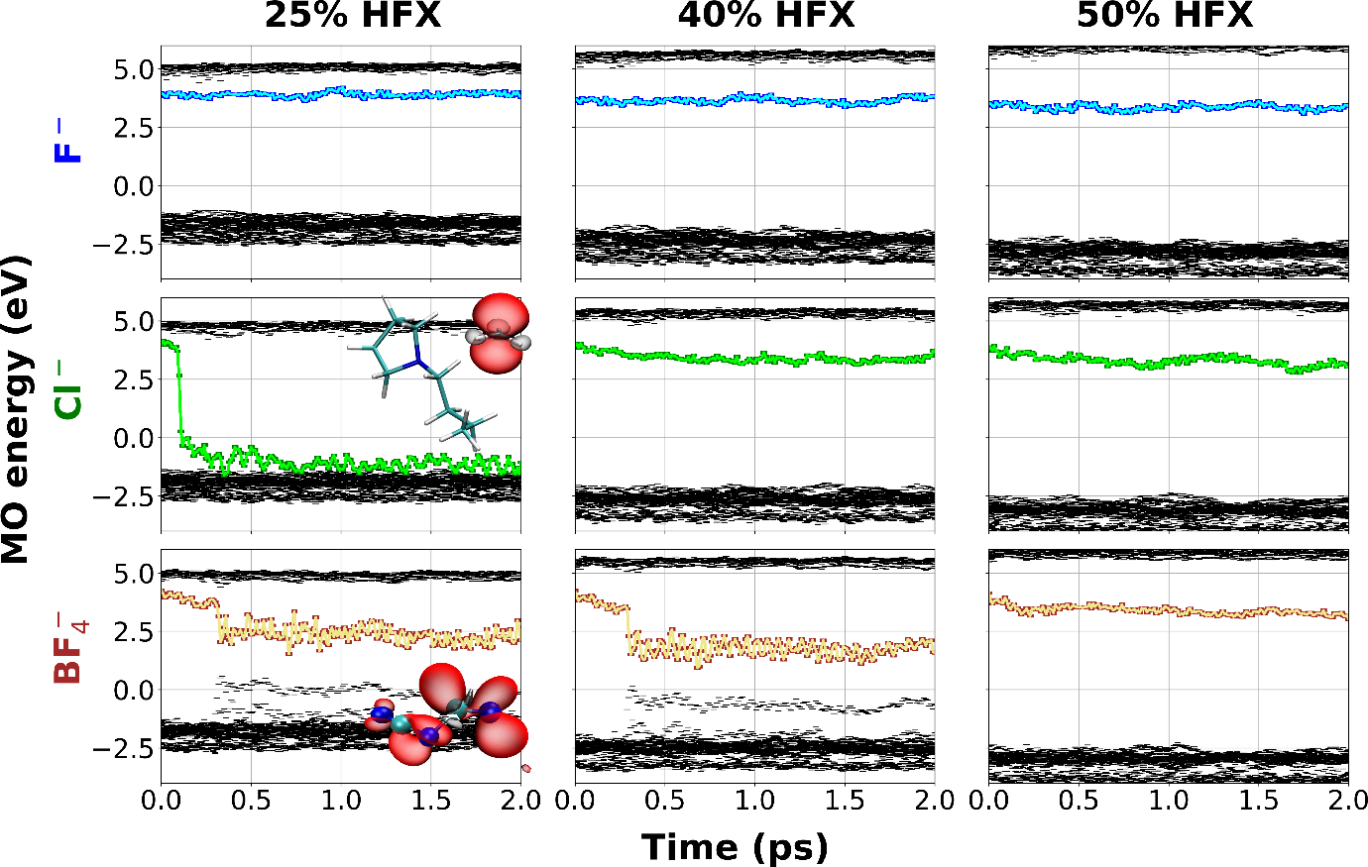
For PBE0-D3 trajectories with different fractions of HFX denoted at the top of each column and for different ansatz for initial electron localization denoted on the left (anions of different size were removed at time zero to induce electron localization at the position of this removal), molecular orbital energy diagrams as a function of time. SOMO depicted in color.

So how do we interpret all of these computational results in the context of what we simplistically called at the beginning of this letter the “electrochemical” (or the anionic reduction) result vs the radiolysis and photolysis results presented here? In prior bulk-phase computational work using simpler techniques such as the PBE functional,^19,20,25,26^ the observation was that the initial electron is, as expected due to delocalization issues in DFT, delocalized. Now, as we systematically vary the amount of HFX, we can confirm that a more delocalized electron appears to be able to quickly “search and find” ionic targets that are lower in SOMO energy than a cavity electron. Instead, a conceivably more realistic, more localized representation of the excess electron can be trapped in cavities and undergo full solvent reorganization to become what in the jargon is called a “solvated” electron. The fully solvated particle-in-a-box-style electron needs to climb and cross barriers to escape its surrounding cationic environment. From the first column in Figure 3, we notice that the reaction of the electron with a cation also appears to be significantly downhill in comparison with the SOMO energy of a cavity electron. In the end, all of these products are reasonably expected: radical CH_3_^·^, radical doubly charged anions, and all of their subsequent downhill reaction products. Seen as a whole, the cavity electron is a higher energy species than other downstream products, but it is kinetically stable in the bulk phase over many hundreds of nanoseconds in this and other ILs.

## Supplementary Material



## Notes

Experimental and computational methods section including the description of pump probe data, pulse radiolysis, classical molecular dynamics, *ab initio* molecular dynamics, and optical spectra calculations (PDF)
