# Supplementary material for: Electrons and Their Multiple Kinetic Fates in an Ionic Liquid
Source: J Am Chem Soc. 2025 Jun 27;147(27):23395–8. doi: 10.1021/jacs.5c07005 (PMC12257543; doi:10.1021/jacs.5c07005)
Supplement: Supplementary file 1 [file ja5c07005_si_001.pdf]

Supporting Information: Electrons and Their Multiple Kinetic Fates in an Ionic Liquid

Hung H. Nguyen,[†] Katie Huber,[‡] Dishan Das,[†] Bichitra Borah,[†] Matthew S. Emerson,[¶] Meghan Knudtzon,[‡] James F. Wishart,[*,¶] David A. Blank,[*,‡] and Claudio J. Margulis[*,†]

†*Department of Chemistry, The University of Iowa, Iowa City, Iowa 52242, United States*

‡*Department of Chemistry, University of Minnesota, Minneapolis, MN 55455, United States*

¶*Chemistry Department, Brookhaven National Laboratory, Upton, NY 11973, United States*

E-mail: wishart@bnl.gov; blank@umn.edu; claudio-margulis@uiowa.edu

S.1 Experimental and Computational Methods

Below we describe our approach to sample handling, pulse radiolysis and optical studies as well as computer simulation studies on the excess electron in $[Pyrr_{1,4}][N(CN)_2]$.

S.1.1 Experimental Section

This section describes our pulse radiolysis experiments, and the new way we analyzed pump-probe data originally collected in reference S1.

S.1.1.1 Pump Probe Data

The pump-probe data for $[Pyrr_{1,4}][N(CN)_2]$ was originally reported in Figure 2 of Knudtzon *et al*,[S1] but has been re-processed for easy comparison with computational results shown in Figure 1 of the main text. The experimental conditions under which these data were collected, including a description of the laser system used and sample handling, were already given in reference S1. In this setup, transient absorption spectra were collected at discrete time delays after the pump pulse initiated excited state dynamics. In the current article, rather than displaying selected spectra at individual time delays, we chose to average spectra over several discrete delays; specifically, 400-600 fs, 1-2 ps, and 2-4 ps. Between 400 and 600 fs, five spectra were averaged and between 1 and 4 ps, 22 spectra were averaged.

S.1.1.2 Pulse Radiolysis

The ionic liquid 1-butyl-1-methyl-pyrrolidinium dicyanamide, $[Pyrr_{1,4}][N(CN)_2]$, used in the pulse radiolysis experiments was purchased from Iolitec and purified using a procedure similar to one previously described.[S1] The sample was first stirred with activated charcoal (DARCO, Sigma-Aldrich, 100 mesh particle size), filtered through a fine glass-frit Buchner funnel (ChemGlass), and its absorption measured by UV-Vis. The onset of absorption shifts to higher energies with the removal of impurities; cycles of stirring and filtering were repeated four times until there was no significant change in the onset. The sample was dried overnight in a vacuum oven at 45 °C, then transferred to an Ar-filled glovebox and placed in a septum-capped, 5 mm path length Suprasil spectrophotometer cuvette prior to experiments.

Pulse radiolysis experiments were carried out at the Laser Electron Accelerator Facility (LEAF) at Brookhaven National Laboratory.[S2] Electron pulse durations were less than 50 ps and typically delivered 11 Gy/pulse. Radiation dosimetry was performed at ambient temperature using the absorbance of the solvated electron in a standard solution (1.0 M NaOH, and 20 vol % ethanol in Millipore DI water) measured at 650 nm (G = 3.48, ε = 20477). A pulsed xenon arc lamp provided analyzing light for detection in the range of 310-1600 nm, while bandpass interference filters were

used to select specific wavelengths (10 nm below 800 nm and 25 nm at 800 nm and above, Edmund Optics). Silicon (300-1000 nm, FND-100Q, Excelitas Technologies) and germanium (900-1600 nm, GEP-600, GPD Optoelectronics Corp) photodiodes were used to detect the selected wavelength. Signals were digitized using a Teledyne LeCroy WaveRunner 640Zi 4 GHz, 8-bit, HDO6104 1 GHz, 12-bit oscilloscope. Data was processed using customized LabVIEW (National Instruments) and Igor Pro (Wavemetrics, Inc.) software routines.

S.1.2 Computational Methodology

Our study is based on multiple different first principles molecular dynamics studies as described in detail in subsections below. To generate well-equilibrated initial conditions for these, we performed classical molecular dynamics (CMD) simulations.

S.1.2.1 Classical Molecular Dynamics

All classical molecular dynamics simulations for initial equilibration were conducted using the GROMACS-5.1.4 software package.[S3–S5] Simulation boxes contained 16 ion pairs, except for one, which had one fewer anion. Initial configuration were generated using the Packmol software.[S6] The table below shows the number of cations and anions in simulated each box.

Table S.1: Composition of Ionic Liquid Systems

	**Cation**	**Anions**				**Systems**
	1-butyl-1-methylpyrrolidinium	dicyanamide	fluoride	chloride	tetrafluoroborate	
No. of Ions	16	15	1			IL Box 1
	16	15		1		IL Box 2
	16	15			1	IL Box 3
	16	15				IL Box 4

The parameters for bonds, angles, dihedrals, and Lennard-Jones terms were adapted from the CL&P and OPLS-AA force fields.[S7–S11] The charges for BF_4^- and the non-bonded parameters for Cl^- were obtained from Padua group's GitHub page, specifically from the il.ff file, accessed on 09/11/2023. Simulation protocols were consistent across all systems. Each system was first

energy-minimized and later equilibrated at 300 K. The equilibration process involved progressively scaling the charges: first, by 1% for 2 ns at 50 bar, followed by 10% for 2 ns at 50 bar, and finally 100% for 2 ns at 1 bar in the constant temperature and pressure (NPT) ensemble. The systems were later subjected to simulated annealing for 10 ns at 1 bar within the same NPT ensemble, where the temperature was gradually ramped from 300 K to 600 K, followed by cooling back to 300 K at full charge. Following the annealing process, a final run was conducted for 10 ns at 300 K, maintaining the same NPT ensemble conditions. The V-rescale thermostat[S12] (0.2 ps time constant) and Berendsen barostat[S13] (1.0 ps time constant) were used during equilibration steps. The Nosé-Hoover thermostat[S14] (0.2 ps time constant) and Parrinello-Rahman barostat[S15] (1.0 ps time constant) were used for the annealing and final run. All the simulations used the MD integrator as coded in GROMACS. The cutoff for all the non-bonded interactions was set to 0.6 nm. Electrostatic interactions were computed using the Particle-mesh Ewald method[S16,S17] with 0.08 nm Fourier spacing and a sixth order interpolation. In each case, a simulation frame was taken from the final run that closely matched the average equilibrium density, and this frame was used as the input file for *ab initio* molecular dynamics (AIMD) simulations.

S.1.2.2 *Ab initio* Molecular Dynamics Studies and Optical Spectra Calculations

AIMD Equilibration

All AIMD simulations were performed using the Quickstep module of the CP2K package[S18] at 300 K in the canonical (NVT) ensemble with a 1 fs timestep and used the Nosé–Hoover thermostat.[S14,S19] The generation of initial frames for these was already described in section S.1.2.1. Starting from these, we performed partial (a few tens of cycles) conjugate gradient energy optimizations in order to bring bond lengths and angles from their classical values to the preferred values for the first principles potential. These structural optimizations and subsequent 20 ps AIMD equilibration runs without an excess electron were at the PBE-D3 level of theory,[S20–S23] and carried out as spin-restricted with a 600 Ry plane-wave cutoff and DZVP basis sets combined with GTH pseudopotentials as coded in CP2K.[S24,S25] Final frames from these PBE-D3 equilibration

runs served as the initial set of coordinates for further AIMD simulations with the excess electron.

Electronic Structure Methods When Including an Excess Electron

Because we suspected that the level of electron localization could influence the early time-dependent structure of the excess electron and its reactivity, we examined various electronic structure methods including the hybrid functional PBE0 with different fractions of HFX (25%, 40%, and 50%), as well as the newly developed DC-r^{2}SCAN method.[S26,S27]

For AIMD runs using the PBE0 method, we employed triple-zeta polarized (TZVP) basis sets with GTH-PBE0 pseudopotentials, a 600 Ry auxiliary plane-wave cutoff, and the D3 correction by Grimme.[S23] To reduce computational cost, the ADMM-TZP basis sets were used to construct auxiliary density matrices via the auxiliary density matrix method (ADMM).[S28] The Schwarz integral screening threshold and the cutoff radius for the truncated Coulomb potential were set to 10^{-8} and 6.0 Å respectively. In the case of the simulations using the DC-r^{2}SCAN method, we used triple-zeta double-polarized (TZV2P) basis sets, GTH-SCAN pseudopotentials, cpFIT3 ADMM basis sets for auxiliary density matrices construction, and a 1200 Ry plane-wave cutoff. In this case, the thresholds for Schwarz integral screening and the cutoff radius for the truncated Coulomb potential were set to 10^{-7} and 6.0 Å respectively. All production PBE0 trajectories were 2 ps in duration and DC-r^{2}SCAN trajectories were 5 ps in duration.

All simulations including an excess electron were run as spin polarized, and they all had zero total charge and an unpaired electron. In the case of our PBE0 calculations this was achieved by removing an anion. Specifically, we attempted to bias the initial location of the excess electron by removing anions of different size (F^-, Cl^-, BF_4^-) providing pre-equilibrated ansatz locations where an excess electron could be favorably surrounded by cations. Instead, from a given set of initial coordinates, we generated two runs using the DC-r^{2}SCAN technique. In this case, instead of biasing the system structurally, we instead managed to generate two different initial density matrix guesses by converging zero-time single point calculations with different plane-wave cuttoff values (1200 Ry and 2000 Ry). Then simulations were run using the 1200 Ry value. In essence, for these runs we were able to easily create different initial conditions for the excess electron at time zero

because it was “dry”; in other words, there was no significantly preferred location pre-solvated by cations.

Optical Spectra Calculations

In all cases presented in Figure 1 of the main text, optical spectra calculations were carried out using TDDFT under the Tamm-Dancoff approximation as coded in CP2K. For these we used the PBE0-D3 scheme with 50% Hartree Fock exchange (HFX) and coordinates derived from one of our DC-r^{2}SCAN simulations in which the result was a cavity electron; the plane-wave cutoff for these was set to 800 Ry. More details of how these data are presented can be found in the caption of Figure 1.

References

(S1) Knudtzon, M. N.; Blank, D. A. Photodetachment and Electron Dynamics in 1-Butyl-1-methyl-pyrrolidinium Dicyanamide. *J. Phys. Chem. B* **2020**, *124*, 9144–9153.

(S2) Wishart, J. F.; Cook, A. R.; Miller, J. R. The LEAF picosecond pulse radiolysis facility at Brookhaven National Laboratory. *Rev. Sci. Instrum.* **2004**, *75*, 4359–4366.

(S3) Hess, B.; Kutzner, C.; van der Spoel, D.; Lindahl, E. GROMACS 4: Algorithms for Highly Efficient, Load-Balanced, and Scalable Molecular Simulation. *J. Chem. Theory Comput.* **2008**, *4*, 435–447.

(S4) Abraham, M. J.; Murtola, T.; Schulz, R.; Páll, S.; Smith, J. C.; Hess, B.; Lindahl, E. GROMACS: High performance molecular simulations through multi-level parallelism from laptops to supercomputers. *SoftwareX* **2015**, *1-2*, 19–25.

(S5) Spoel, D. V. D.; Lindahl, E.; Hess, B.; Groenhof, G.; Mark, A. E.; Berendsen, H. J. C. GROMACS: Fast, flexible, and free. *J. Comput. Chem.* **2005**, *26*, 1701–1718.

(S6) Martínez, L.; Andrade, R.; Birgin, E. G.; Martínez, J. M. PACKMOL: A package for build-

ing initial configurations for molecular dynamics simulations. *J. Comput. Chem.* **2009**, *30*, 2157–2164.

(S7) Rizzo, R. C.; Jorgensen, W. L. OPLS All-Atom Model for Amines: Resolution of the Amine Hydration Problem. *J. Am. Chem. Soc.* **1999**, *121*, 4827–4836.

(S8) Jorgensen, W. L.; Maxwell, D. S.; Tirado-Rives, J. Development and Testing of the OPLS All-Atom Force Field on Conformational Energetics and Properties of Organic Liquids. *J. Am. Chem. Soc.* **1996**, *118*, 11225–11236.

(S9) Kaminski, G.; Jorgensen, W. L. Performance of the AMBER94, MMFF94, and OPLS-AA Force Fields for Modeling Organic Liquids. *J. Phys. Chem.* **1996**, *100*, 18010–18013.

(S10) Canongia Lopes, J. N.; Pádua, A. A. H. Molecular Force Field for Ionic Liquids III: Imidazolium, Pyridinium, and Phosphonium Cations; Chloride, Bromide, and Dicyanamide Anions. *J. Phys. Chem. B* **2006**, *110*, 19586–19592.

(S11) Price, M. L. P.; Ostrovsky, D.; Jorgensen, W. L. Gas-phase and liquid-state properties of esters, nitriles, and nitro compounds with the OPLS-AA force field. *J. Comput. Chem.* **2001**, *22*, 1340–1352.

(S12) Bussi, G.; Donadio, D.; Parrinello, M. Canonical sampling through velocity rescaling. *J. Chem. Phys.* **2007**, *126*, 014101.

(S13) Berendsen, H. J. C.; Postma, J. P. M.; Van Gunsteren, W. F.; Dinola, A.; Haak, J. R. Molecular dynamics with coupling to an external bath. *J. Chem. Phys.* **1984**, *81*, 3684–3690.

(S14) Nosé, S. A unified formulation of the constant temperature molecular dynamics methods. *J. Chem. Phys.* **1984**, *81*, 511–519.

(S15) Parrinello, M.; Rahman, A. Polymorphic transitions in single crystals: A new molecular dynamics method. *J. Appl. Phys.* **1981**, *52*, 7182–7190.

(S16) Darden, T.; York, D.; Pedersen, L. Particle mesh Ewald: An N·log(N) method for Ewald sums in large systems. *J. Chem. Phys.* **1993**, *98*, 10089–10092.

(S17) Essmann, U.; Perera, L.; Berkowitz, M. L.; Darden, T.; Lee, H.; Pedersen, L. G. A smooth particle mesh Ewald method. *J. Chem. Phys.* **1995**, *103*, 8577–8593.

(S18) Kühne, T. D.; Iannuzzi, M.; Del Ben, M.; Rybkin, V. V.; Seewald, P.; Stein, F.; Laino, T.; Khaliullin, R. Z.; Schütt, O.; Schiffmann, F.; Golze, D.; Wilhelm, J.; Chulkov, S.; Bani-Hashemian, M. H.; Weber, V.; Borštnik, U.; Taillefumier, M.; Jakobovits, A. S.; Lazzaro, A.; Pabst, H.; Müller, T.; Schade, R.; Guidon, M.; Andermatt, S.; Holmberg, N.; Schenter, G. K.; Hehn, A.; Bussy, A.; Belleflamme, F.; Tabacchi, G.; Glöß, A.; Lass, M.; Bethune, I.; Mundy, C. J.; Plessl, C.; Watkins, M.; VandeVondele, J.; Krack, M.; Hutter, J. CP2K: An electronic structure and molecular dynamics software package - Quickstep: Efficient and accurate electronic structure calculations. *J. Chem. Phys.* **2020**, *152*.

(S19) Martyna, G. J.; Klein, M. L.; Tuckerman, M. Nosé–Hoover chains: The canonical ensemble via continuous dynamics. *J. Chem. Phys.* **1992**, *97*, 2635–2643.

(S20) Perdew, J. P.; Burke, K.; Ernzerhof, M. Generalized Gradient Approximation Made Simple. *Phys. Rev. Lett.* **1996**, *77*, 3865–3868.

(S21) Perdew, J. P.; Burke, K.; Ernzerhof, M. Perdew, Burke, and Ernzerhof Reply:. *Phys. Rev. Lett.* **1998**, *80*, 891–891.

(S22) Zhang, Y.; Yang, W. Comment on "Generalized Gradient Approximation Made Simple". *Phys. Rev. Lett.* **1998**, *80*, 890–890.

(S23) Grimme, S.; Antony, J.; Ehrlich, S.; Krieg, H. A consistent and accurateab initioparametrization of density functional dispersion correction (DFT-D) for the 94 elements H-Pu. *J. Chem. Phys.* **2010**, *132*.

(S24) Goedecker, S.; Teter, M.; Hutter, J. Separable dual-space Gaussian pseudopotentials. *Phys. Rev. B* **1996**, *54*, 1703–1710.

(S25) Hartwigsen, C.; Goedecker, S.; Hutter, J. Relativistic separable dual-space Gaussian pseudopotentials from H to Rn. *Phys. Rev. B* **1998**, *58*, 3641–3662.

(S26) Belleflamme, F.; Hutter, J. Radicals in aqueous solution: assessment of density-corrected SCAN functional. *Phys. Chem. Chem. Phys.* **2023**, *25*, 20817–20836.

(S27) Furness, J. W.; Kaplan, A. D.; Ning, J.; Perdew, J. P.; Sun, J. Accurate and Numerically Efficient r2SCAN Meta-Generalized Gradient Approximation. *J. Phys. Chem. Lett.* **2020**, *11*, 8208–8215.

(S28) Guidon, M.; Hutter, J.; VandeVondele, J. Auxiliary Density Matrix Methods for Hartree-Fock Exchange Calculations. *J. Chem. Theory Comput.* **2010**, *6*, 2348–2364.
